# Synthesis and characterization of a novel magnetic chitosan–nickel ferrite nanocomposite for antibacterial and antioxidant properties

**DOI:** 10.1038/s41598-023-42974-6

**Published:** 2023-09-22

**Authors:** Samira Shokri, Nabi Shariatifar, Ebrahim Molaee-Aghaee, Gholamreza Jahed Khaniki, Parisa Sadighara, Mohammad Ali Faramarzi, Mansoureh Mohammadi, Alieh Rezagholizade-shirvan

**Affiliations:** 1 Department of Environmental Health, Food Safety Division, School of Public Health, Tehran University of Medical Sciences, Tehran, Iran; 2 Department of Pharmaceutical Biotechnology, Faculty of Pharmacy, Tehran University of Medical Sciences, Tehran, Iran; 3 Department of Food Science and Technology, Faculty of Nutrition Science and Food Technology, National Nutrition and Food Technology Research Institute, Shahid Beheshti University of Medical Sciences, Tehran, Iran; 4 Department of Food Science and Technology, Neyshabur University of Medical Sciences, Neyshabur, Iran

**Keywords:** Environmental sciences, Chemistry

## Abstract

A novel nanomagnet modified with nickel ferrite nanoparticles (NPs) coated with hybrid chitosan (Cs–NiFe_2_O_4_) was synthesized using the co-precipitation method. The resulting nanomagnets were characterized using various techniques. The size of the nanomagnetic particles was estimated to be about 40 nm based on the transmission electron microscopy (TEM) image and X-ray diffraction analysis (XRD) pattern (using the Debye–Scherrer equation). Scanning electron microscopy (SEM) images indicated that the surface of Cs–NiFe_2_O_4_ NPs is flatter and smoother than the uncoated NiFe_2_O_4_ NPs. According to value stream mapping (VSM) analysis, the magnetization value of Cs–NiFe_2_O_4_ NPs (17.34 emu/g) was significantly lower than NiFe_2_O_4_ NPs (40.67 emu/g). The Cs–NiFe_2_O_4_ NPs indicated higher antibacterial properties than NiFe_2_O_4_ NPs and Cs. The minimum inhibitory concentrations of Cs–NiFe_2_O_4_ NPs against *S. aureus* and *E. coli* were 128 and 256 mg/mL, respectively. Antioxidant activity (evaluated by 2,2-diphenyl-1-picrylhydrazyl (DPPH) scavenging test) for NiFe_2_O_4_ NPs and Cs–NiFe_2_O_4_ NPs at the concentration of 100 µg/mL were 35% and 42%, respectively. Consequently, the synthesized Cs–NiFe_2_O_4_ NPs can be proposed as a viable material for biomedical applications.

## Introduction

Recently, new studies have focused on producing spinel ferrite nanocrystals with size-dependent characteristics and a high surface-to-volume ratio^1^. Among the spinel ferrite nanomaterials, NiFe_2_O_4_ NPs are of significant importance due to the inverse configuration of spinel and ferrimagnetism caused by anti-parallel spins between Fe^3^^+^ ions in tetrahedral sites and Ni^2^^+^ ions in octahedral sites^2,3^. The magnetic properties of NPs are influenced by the size, shape, degree of crystallinity, shape, and coating around the nanoparticles^4^. The high surface energy and strong interactions between dipoles significantly increase the tendency of NiFe_2_O_4_ NPs to aggregate. Therefore, it is necessary to cover the nanoparticles’ surfaces with organic biocompatible and biodegradable materials^2,3^.

Coatings can be made of various materials, including chitosan^5,6^, starch, polyethylene glycol, polyvinyl alcohol^5,7^, dextran^5,7^, and oleic acid^8^. Chitosan, a cationic linear biopolymer, is known for its unique properties, e.g., availability, safety, hydrophilicity, biocompatibility, biodegradability, and antimicrobial activity. It is widely used in biomedical fields^9^. Besides, it can be utilized as a basic material for synthesizing non-toxic biocompatible films with strong mechanical strength and antibacterial potential. Chitosan exhibits a broad-spectrum antibacterial activity against Gram-positive and Gram-negative bacteria through multiple mechanisms, including disrupting the bacterial cell membrane and interacting with bacterial DNA, leading to the inhibition of DNA replication and protein synthesis^10,11^. Chitosan-based biosensors can create hydrogen bonds between the hydrogen atom of their amino groups and the oxygen atom of ferrite^12^. Recent cytotoxicity evaluations have shown that NiFe_2_O_4_ nanoparticles, coated with or without chitosan, exhibit non-cytotoxic behavior^12^.

Biomedical materials have shown immense potential in anticancer, antioxidant, and antibacterial applications, combating oxidative stress and bacterial infections^13,14^. They can scavenge free radicals, mitigate oxidative damage, and promote overall health^15^. These materials also possess antimicrobial properties, inhibiting bacterial growth and reducing the risk of infections^16,17^. Biomedical materials that combine antioxidant and antibacterial activity offer a dual-action approach to healing and preventing complications^18^. However, challenges such as biocompatibility and optimization of properties need to be addressed^19^. Therefore, collaborative efforts are crucial for advancing the field and revolutionizing healthcare.

Pathogenic bacteria such as *S. aureus* and *E. coli* are the most significant causes of food- and water-borne diseases^20^. These bacteria are often found in biofilms, where they develop in a polymeric extracellular matrix that surrounds the bacterial cells and acts as a diffusion barrier by trapping and degrading antibiotic molecules^21,22^. These polymeric substances can form well-organized networks impermeable to small molecules^23,24^. Nanomaterials can effectively combat and prevent microbial resistance^25^. The antibacterial nanoparticles have several advantages over conventional antibiotics, including prevention of antibiotic resistance mechanisms, rupture of bacterial membranes, simultaneous attack through multiple approaches to bacteria, and effective performance as antibiotic carriers^26,27^. Researchers have looked for metal or metal oxide nanoparticles to kill bacteria while avoiding common antibiotic resistance mechanisms such as permeability regulation, biofilm formation, multidrug efflux pumps, antibiotic degradation, and gene changes^28,29^.

The chief limitation of common preparation procedures is particle accumulation, which restricts their scope of applications. Co-precipitation is an efficient approach to produce the NiFe_2_O_4_ NPs with Cs coating. To our knowledge, no investigation has been conducted on the antioxidant and antibacterial activities of Cs–NiFe_2_O_4_ NPs. This study aimed to synthesize Cs-based NiFe_2_O_4_ nanoparticles using the co-precipitation method. Hybridization of chitosan with NiFe_2_O_4_ NPs was performed to explore the potential of chitosan to improve the overall efficiency and antibacterial and antioxidant properties of NiFe_2_O_4_ NPs. In addition, the structural, textural, morphological, antibacterial, and antioxidant characteristics of synthesized nanoparticles were evaluated.

## Experimental section

### Materials and bacterial strains

Chitosan (Low Mw, 50,000–190,000 KDa), acetic acid (≥ %98), FeCl_3_–6H_2_O, NiCl_2_–6H_2_O, NaOH, Muller Hinton Agar (MHA), and oleic acid were obtained from Merck Company (Darmstadt, Germany).

The *E. coli* (ATCC 8739) and *S. auras* (ATCC 6537) were purchased from the Iranian Research Organization for Science and Technology (Tehran, Iran). For activation of bacteria, the lyophilized cultures were incubated consecutively twice in a TSB medium at 37 °C for 24 h.

### Production of Chitosan-coated NiFe_2_O_4_ nanoparticles

#### Preparation of NiFe_2_O_4_ nanoparticles

The synthesis of NiFe_2_O_4_ NPs was performed through the co-precipitation technique^30^. In summary, FeCl_3_.6H_2_O (0.5 g/10 mL) was mixed with NiCl_2_.6H_2_O (1.5 g/50 mL) and stirred for 30 min at 60 °C. The pH was adjusted to ~ 11 by NaOH (0.2 M). Then, 0.01 mL oleic acid, as a surfactant, was added to the mixture. The mixture was heated to 80 °C and stirred for 6 h. After centrifuging (5424 R, Eppendorf AG, Germany), distilled water and ethanol were used to rinse the precipitates. The resulting dark brown precipitates were dried at 25 °C for 20 h, transferred to a tube furnace, and calcined for three hours at 600 °C under the air environment^31^.

#### Synthesis of Cs–NiFe_2_O_4_

Chitosan solution (1%, w/v) was prepared by dissolving Cs powder in acetic acid solution (95% v/v) by stirring. NiFe_2_O_4_ NPs were added to distilled water and stirred for 15 min. The nanoparticle dispersion was further sonicated (km-GT, Korea) for 15 min. Subsequently, the Cs solution was added to the NP suspension under slow stirring and kept at 50 °C for about 3 h. The resulting solution was sonicated for ~ 30 min, homogenized by stirring and centrifuged for 10 min. Brown precipitates were separated by filtering, washed, and dried at 25 °C for 20 h^31–33^.

### Characterization of prepared materials

The X-ray diffraction (XRD) technique was carried out by an X-ray diffractometer (Tongda TD-3700, Germany) with CuKα radiation, a nickel monochromator, and a detector operating at 40 kV and 30 mA. The XRD pattern was recorded at 2θ = 5–80° by a scanning speed of 0.04°/min at 25 °C. The Fourier transform infrared spectra (FTIR) of the produced NPs were documented at the range of 500–4000 cm^-1^ at a resolution of 4 cm^-1^ by a Bruker Tensor 27 FTIR spectrophotometer. The morphological investigation was carried out by scanning electron microscopy (SEM, MIRA3-LUM, Czech) and transmission electron microscopy (TEM, EM Philips EM 208S). The magnetic feature was detected utilizing a vibrating sample magnetometer (VSM, Lakeshore).

### Antibacterial activities

The antibacterial effects of green synthesized Cs–NiFe_2_O_4_ NPs (8, 16, 32, 64, 128, 256, and 512 mg/mL) on *S. aureus* and *E. coli* bacteria were evaluated using the agar well-diffusion technique on Muller Hinton agar^34^. Initially, the surface of the MHA plate was inoculated with bacteria. Afterward, a well with 6 mm diameter was placed on the inoculated plates. The diameter of inhibition zones was measured after 24 h incubation at 37 °C. Similar evaluations were done on the positive control (MHA plate inoculated with test bacteria) and the negative control (uninoculated MHA plate with wells on which). The minimum inhibitory concentration (MIC) and minimum bactericidal concentration (MBC) of produced NPs were investigated by the broth micro-dilution technique^35–37^. In summary, 100 μL of bacterial suspension containing 10^8^ CFU/mL of test bacteria was added to 100 μL of the NP dilutions in the 96-well microtiter plates containing Muller Hinton broth. The plates were incubated at 37 °C for 24 h. Analogous examinations were carried out as the negative control (nanoparticle dilution plus the culture medium) and the positive control (bacterial suspension plus the culture medium). Then, 100 µL of each well was inoculated on Muller Hinton agar, and the plates were incubated at 37 °C for 24 h. The lowest concentration without turbidity in wells or growth on plates were considered MIC and MBC, respectively.

### Antioxidant activities

The antioxidant potency of synthesized NPs was evaluated by the DPPH scavenging method^38^. First, 100 μL of NPs was added to 1 mL of an ethanolic DPPH solution (0.1 mM ) and stored under darkness at ambient temperature for 60 min. The absorbance (A) was recorded at 517 nm against ethanolic DPPH solution as blank using a UV spectrophotometer. The antioxidant property was calculated according to the following Equation:

1 $$ {\text{Antioxidant activity }}\left( {\text{\% }} \right){ } = \frac{{A_{blank} - { }A_{sample} }}{{A_{blank} }} \times 100. $$

### Statistical analysis

The data obtained from three replications were analyzed using SPSS software by One-way ANOVA followed by Duncan's multiple range tests (α = 0.05), and the results were expressed as mean ± standard deviation.

## Results and discussion

### Characterization

#### XRD analysis

**Figure 1:**
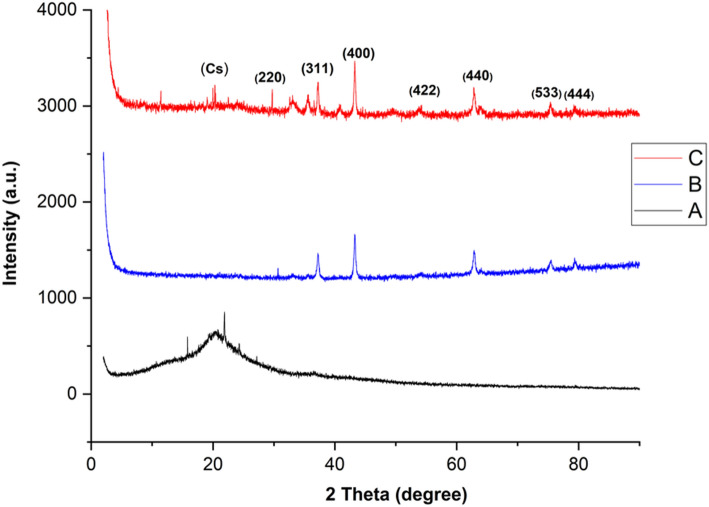
The X-ray diffraction patterns of Cs (**A**), NiFe_2_O_4_ NPs (**B**), and Cs–NiFe_2_O_4_ NPs (**C**).

The crystalline structure of chitosan polymer and uncoated and chitosan-coated NiFe_2_O_4_ nanoparticles was determined by X-ray diffraction technique (Fig. 1). The nanocomposites’ particle size can be calculated using the Debye–Scherrer equation:

2 $$ {\text{D}} = \frac{{{\text{K}}\uplambda }}{{\upbeta {\text{Cos}}\uptheta }} $$

where D is the nanoparticle size (nanometers), K is a constant, λ is the X-ray wavelength (nanometers), β is the width at half of the peak height, and θ is the diffraction angle.

The characteristic peaks of chitosan appeared at 2θ angles equal to 10 and 20.091° (Fig. 1A)^39^. The size of chitosan was found to be about 145 nm. The XRD pattern of NiFe_2_O_4_ NPs (Fig. 1B) corresponds to the XRD spectrum of JCPDS standard card no. 10–0325^40,41^ The peaks at 2θ angles of 30.12°, 37.23°, 43.26°, 54.32°, 63.85°, 75.40°, 80.02° represent (222), (311), (400), (422), (440), (533), and (444) crystal planes, respectively. The observed peaks confirm the complete crystallization of nickel ferrite nanoparticles. The three distinct peaks at 43.26°, 63.85°, and 75.40° indicate the presence of a face-centered cubic structure of NiFe_2_O_4_. The patterns are consistent with the standard data indexed in JCPDS card no. 38–0419^31,39,42^. The size of NiFe_2_O_4_ NPs was estimated to be about 30 nm.

Three characteristic peaks of nickel ferrite nanoparticles were also identified in the XRD pattern of Cs–NiFe_2_O_4_ NPs at lower intensities (Fig. 1C). Similar results have been reported by Zhong-ai^43^ and Sivagurunathan^44^. In addition, the chitosan peaks have a weak intensity in the XRD spectrum of Cs–NiFe_2_O_4_ NPs. It can be justified by disrupting the well-crystalline linear structure of chitosan due to interaction with various monomers. These results confirmed the successful coating of nanoparticles with Cs. Analogous findings have been obtained by Zhang et al.^43^ and Zou et al.^45^ It seems that amine and hydroxyl groups on the polymeric structure of chitosan interact non-covalently with nanoparticles. The size of the prepared composite was estimated to be 40 nm. A reduction in the size was exhibited when forming the composite compared to Cs, leading to an increase in the surface area and activity.

#### FTIR analysis

**Figure 2:**
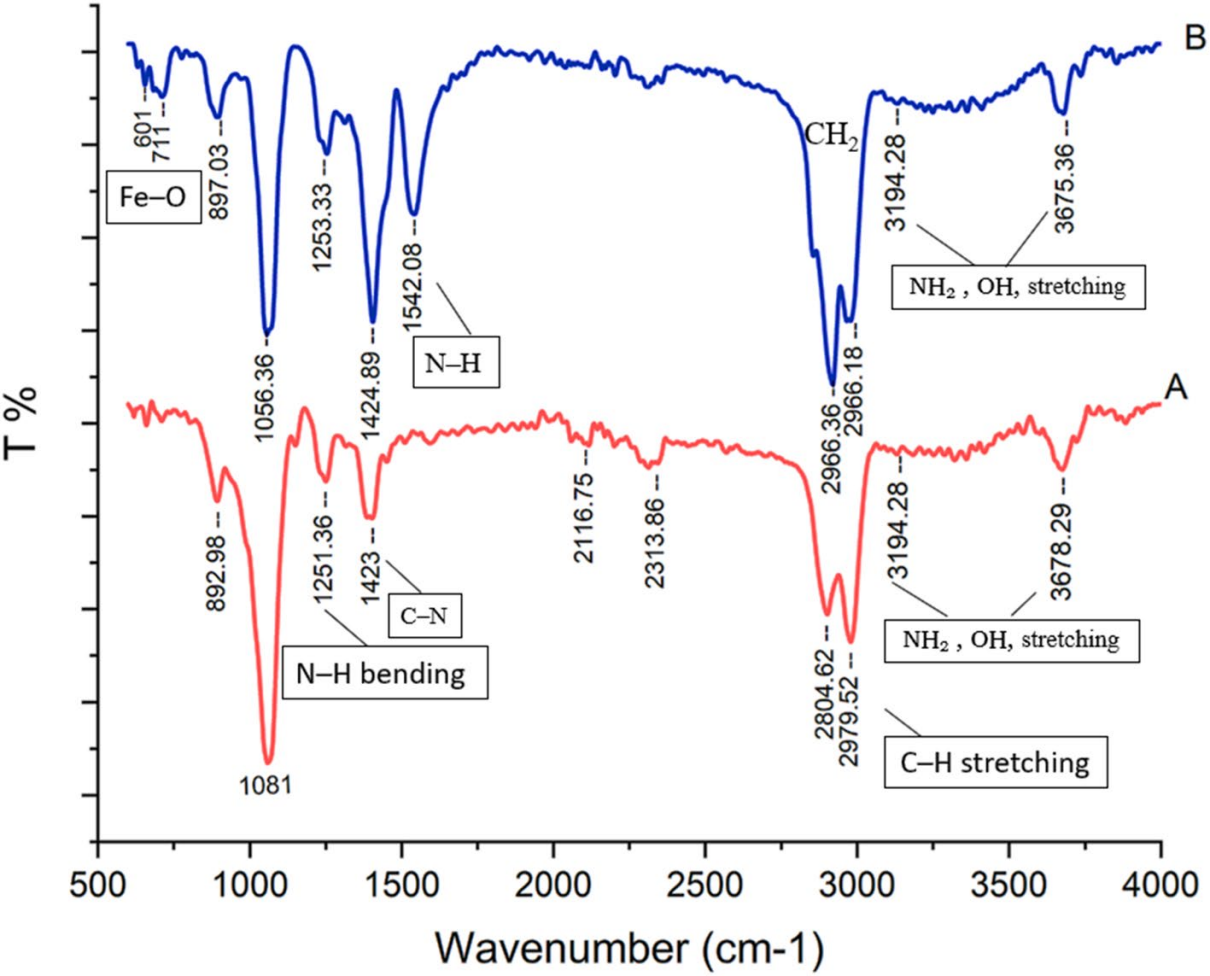
FTIR spectra of Cs (**A**) and Cs–NiFe_2_O_4_ NPs (**B**).

Figure 2 illustrates the FTIR spectra of the samples. The Cs spectrum (Fig. 2A) displays peaks at the wavenumber range of 3194–3678 cm^-1^, which can be ascribed to N–H and O–H stretching vibrations. Moreover, the peaks at 2979 cm^-1^, 1423 cm^-1^, and 1251 cm^-1^ are associated with C–H stretching, C–N carboxylic vibrations of the glycoside ring, and N–H bending vibrations, respectively. FTIR spectrum of Cs–NiFe_2_O_4_ NPs (Fig. 2B) demonstrates a strong peak in 601 cm^-1^ and 711 cm^-1^, which can be attributed to the Fe–O stretching vibration of tetrahedral sites of NiFe_2_O_4_ NPs. The absorption band at 1056 cm^-1^ can be related to sulfate ions adsorbed on the surface of NiFe_2_O_4_ nanoparticles. The bending vibrations of N–H groups are observed at 1542 cm^-1^. The appearance of two peaks at 2968 cm^-1^ and 2919 cm^-1^ corresponded to the stretching vibration of aliphatic hydrogen (–CH_2_). Besides, the bands at 3194 cm^-1^ and 3678 cm^-1^ originated from the stretching vibration of O–H groups^31,46–48^.

These observations can be attributed to an affinity between the negative charge of nickel ferrite nanoparticles and the positive charge of chitosan, which enables the coating of NiFe_2_O_4_ nanoparticles by Cs through electrostatic interactions and chemical reactions. Analogous results were obtained for coating iron oxide nanoparticles with chitosan^9^.

#### TEM analysis

**Figure 3:**
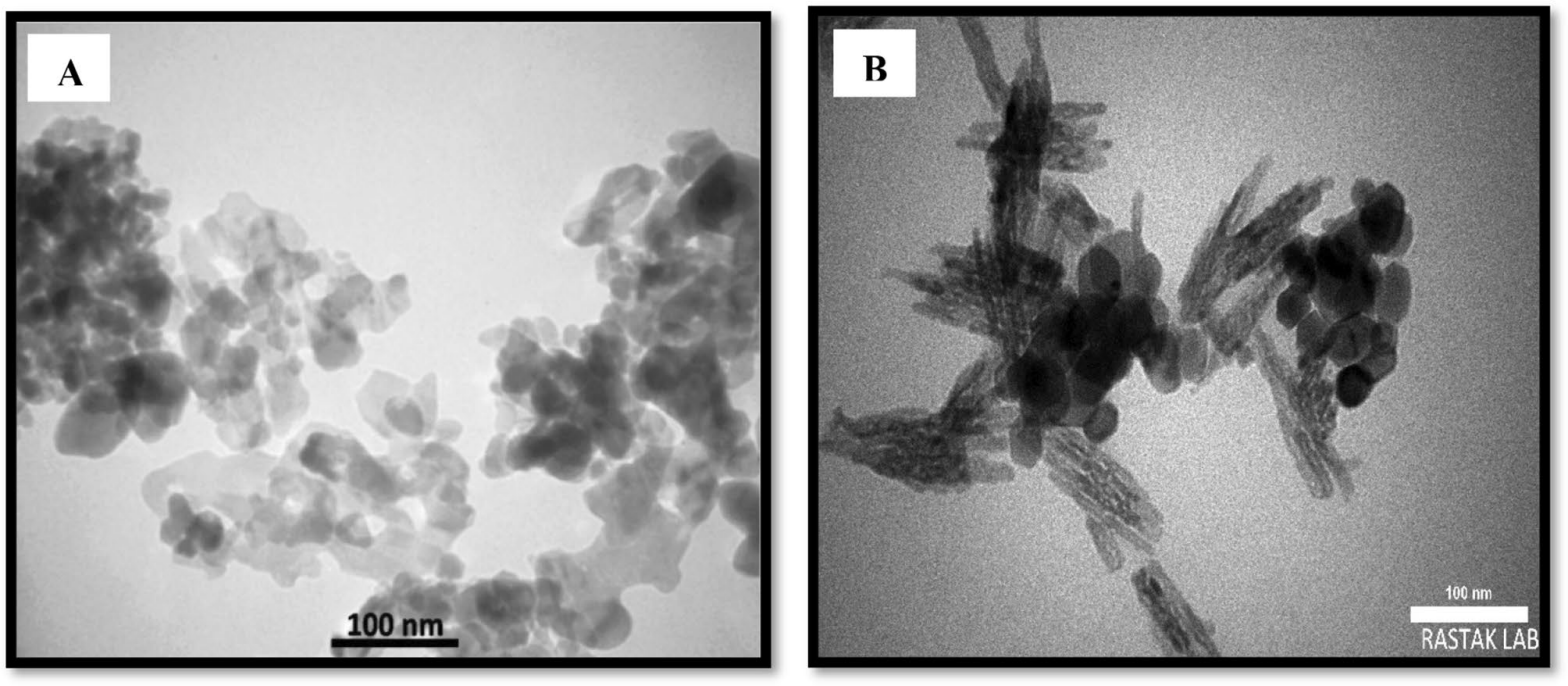
TEM images of NiFe_2_O_4_ NPs (**A**) and Cs–NiFe_2_O_4_ NPs (**B**).

The micromorphology of NiFe_2_O_4_ and Cs–NiFe_2_O_4_ nanoparticles was evaluated via the TEM, and the results were indicated in Fig. 3. According to Fig. 3A, NiFe_2_O_4_ NPs uncoated chitosan only have particle core with a deep color, but in Fig. 3B the Cs–NiFe_2_O_4_ NPs are uniform, spherical in shape, and almost monodisperse. It illustrates the particle core with a deeper color in the magnetic nanoparticles coated with chitosan. Similar results were obtained by Ramezani et al.^49^ for Cs–NiFe_2_O_4_ NPs. The grain size (40 nm) obtained from the TEM images is consistent with that calculated by the Debye–Scherrer equation from the XRD patterns.

#### SEM analysis

**Figure 4:**
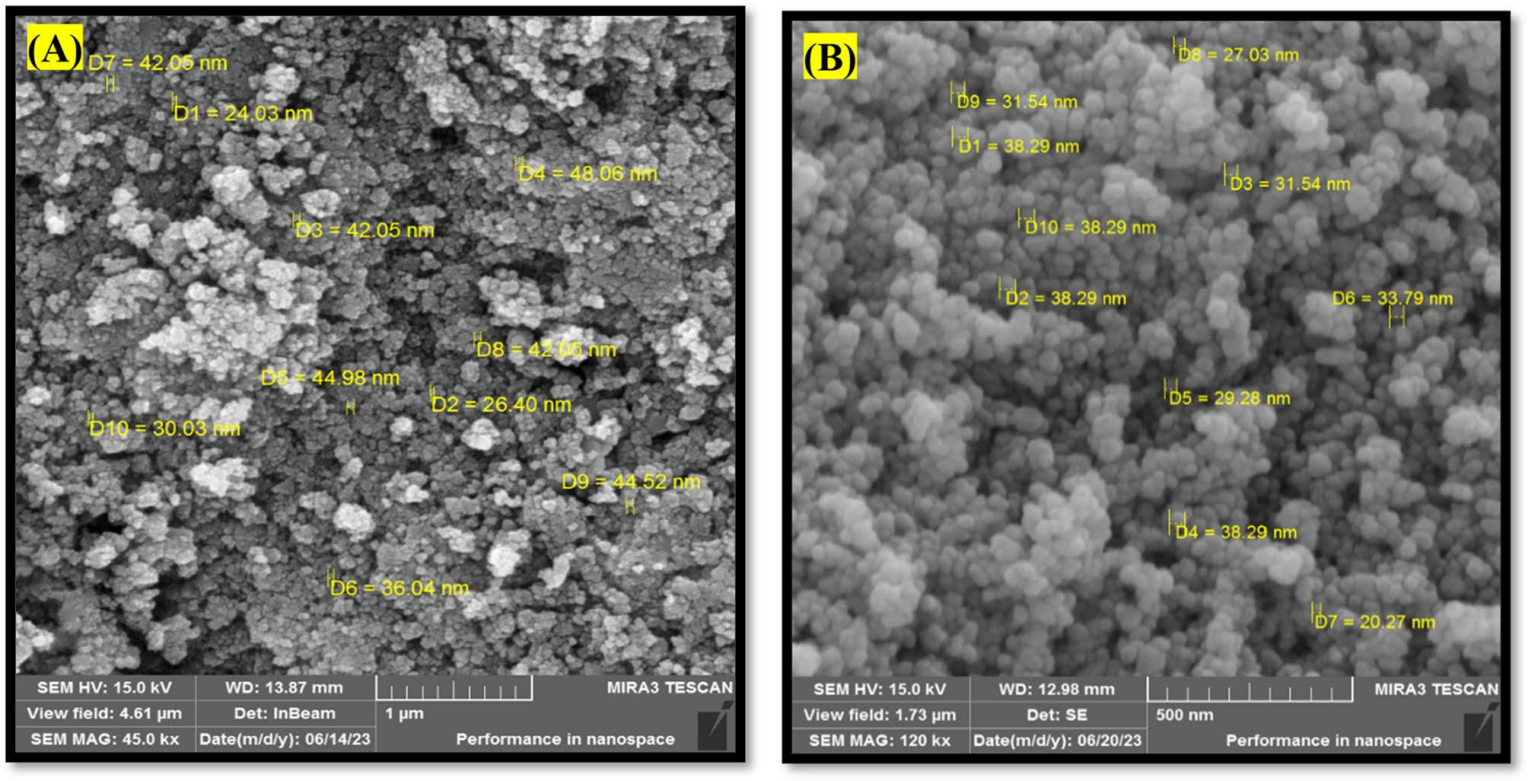
SEM images of NiFe_2_O_4_ NPs (**A**) and Cs–NiFe_2_O_4_ NPs (**B**).

The morphology of NiFe_2_O_4_ and Cs–NiFe_2_O_4_ nanoparticles was investigated using SEM (Fig. 4). Substantial accumulation of nickel ferrite nanoparticles was observed in their natural state (Fig. 4A). An increase in kinetic energy causes the particles to become unstable, leading to a stronger tendency to aggregate and a subsequent increment of the particle size. This phenomenon is related to a decrease in the surface energy of the particles, which is induced by temperature enhancement. According to Fig. 4B, the surface of Cs–NiFe_2_O_4_ NPs is flatter and smoother than the uncoated NPs. The role of chitosan in preventing particle clumping was determined in various dimensions, and the size of NiFe_2_O_4_ nanoparticles was determined to be less than 30 nm. NiFe_2_O_4_ nanoparticles completely dispersed within the polymeric shell of Cs, indicating successful incorporation of NiFe_2_O_4_ NPs in the Cs matrix. These results were consistent with others^7,31,50^.

#### VSM analysis

**Figure 5:**
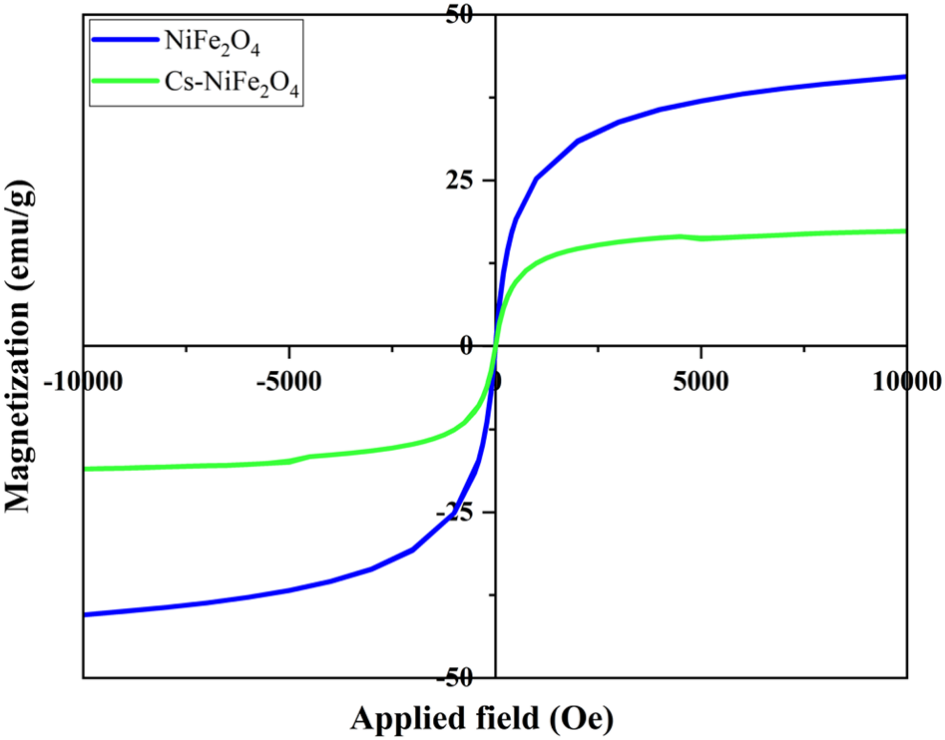
Magnetic hysteresis loop of NiFe_2_O_4_ NPs, and Cs–NiFe_2_O_4_ NPs.

The magnetic properties of Cs–NiFe_2_O_4_ NPs were investigated via hysteresis curves provided by VSM, which revealed zero coercivity and remanence, confirming the super-magnetism of the nanocomposite. The NiFe_2_O_4_ NPs indicated a considerable degree of magnetization (~ 40.67 emu/g). However, when chitosan was incorporated into NiFe_2_O_4_ NPs, the magnetization value significantly decreased to 17.34 emu/g (Fig. 5). This may be due to the presence of additional materials within the nanocomposites, which could influence the magnetic properties of the NiFe_2_O_4_ NPs^51,52^. Moreover, the nanocomposites could easily be separated from aqueous solutions. The super-magnetism and easy separation present a promising material for various industrial purposes. Therefore, Cs–NiFe_2_O_4_ NPs have the potential to contribute to the field of materials science.

### Antibacterial activities

**Figure 6:**
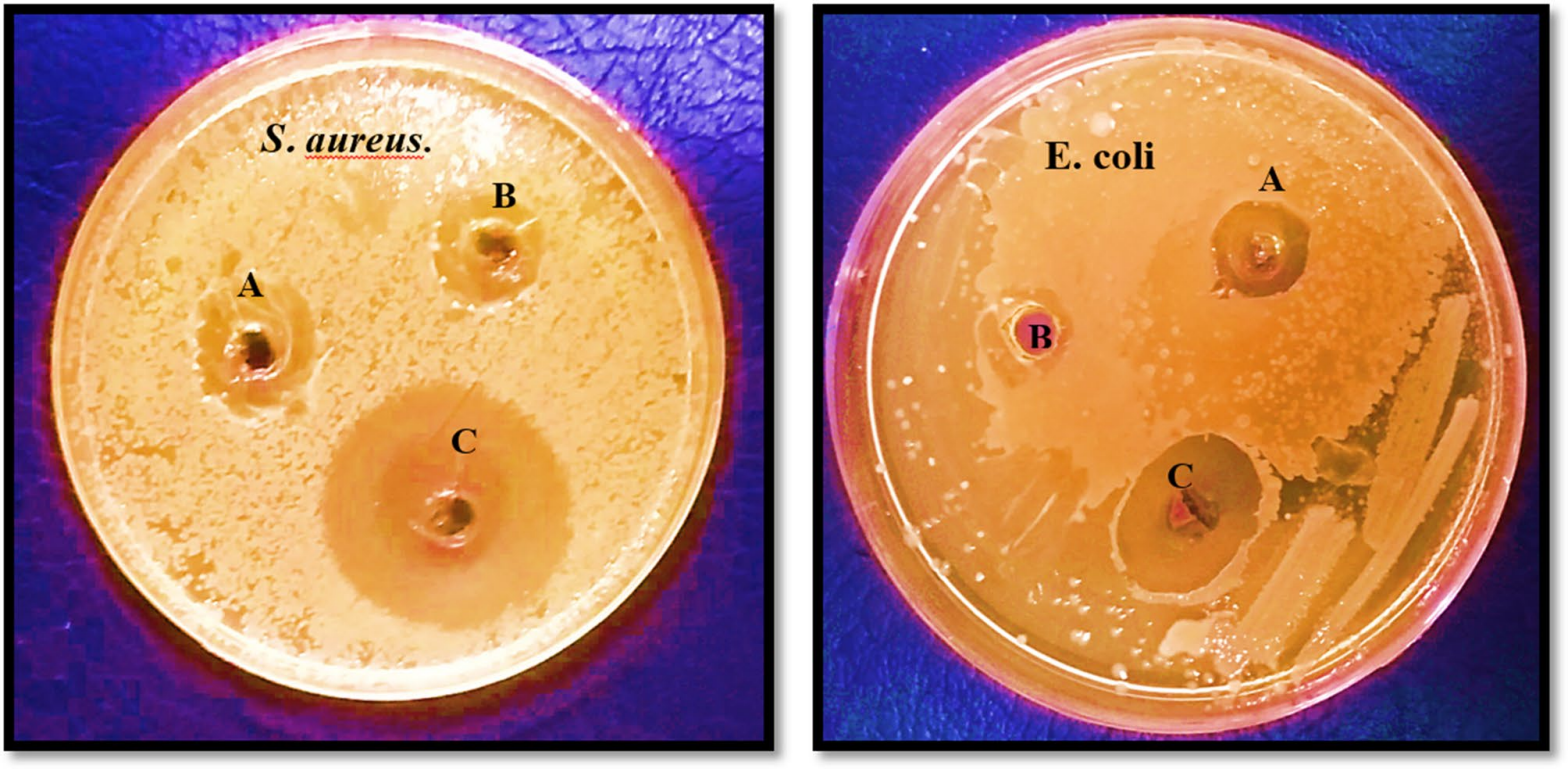
Antibacterial activity of Cs (**A**), NiFe_2_O_4_ NPs (**B**), and Cs–NiFe_2_O_4_ NPs.

Two widespread pathogenic bacterial species, *E. coli* (Gram-negative) and *S. aureus* (Gram-positive), were selected to investigate the antibacterial potency of synthesized nanoparticles. It is imperative to produce effective nano-based materials to reduce the growth and activity of these potentially harmful bacteria in the food and water systems. According to the literature, chitosan has a broad range of antimicrobial activity^53,54^. Antibacterial effects of NiFe_2_O_4_ NPs on *S. aureus* and *E. coli* have also been documented^55,56^. The antibacterial potential of Cs–NiFe_2_O_4_ NPs was significantly higher than that of NiFe_2_O_4_ NPs and Cs at the same concentration (512 µg/mL), possibly due to the synergistic effects between Cs and NiFe_2_O_4_ NPs (Fig. 6). The MIC and MBC of the Cs–NiFe_2_O_4_ NP_S_ against the two pathogenic microorganisms are presented in Table 1. The data revealed that the nanoparticles exhibited higher antibacterial activity on Gram-positive bacteria than Gram-negative bacteria, and *S. aureus* (MIC = 128 mg/mL) is more sensitive to nanoparticles than *E. coli* (MIC = 256 mg/mL). It can be ascribable to the outer lipopolysaccharide layer of gram-negative bacteria, which protects them against nanoparticles. In agreement, Vázquez-Olmos A et al. reported higher antibacterial potency of NiFe_2_O_4_ NPs against *S. aureus* (gram-positive) than that of *P. aeruginosa* (gram-negative)^57^. Table 2 represents the diameters of the inhibition area obtained from the agar-well diffusion assay. The synthesized nanoparticles did not exert antibacterial activity on the bacteria at concentrations below MIC levels. However, at higher concentrations, they indicated acceptable antimicrobial efficiency proportional to the nanoparticle concentration. Similarly, Cs-ZnO NPs illustrated significant antibacterial potency against Escherichia coli^58^. Several studies have described the antimicrobial mechanism of action of Cs and derivatives. The commonly accepted approach is electrostatic interactions between the positively charged amino groups of glucosamine and the negatively charged bacterial cell membrane, leading to significant alterations in membrane permeability. This, in turn, results in an osmotic imbalance and the expulsion of intracellular substances, ultimately causing the cell's death^59–61^. Also, It has been found that the type of bacteria, the size, shape, concentration, and type of nanoparticles, as well as the physicochemical conditions of the reaction medium, affect bacterial sensitivity to metal nanoparticles^62^. Therefore, a decrease in the size of Cs coated on the NiFe_2_O_4_ NP surface leads to an increase in the surface area of Cs for interacting with the cell membranes of bacteria and its antibacterial activity. A comparison of the antibacterial activity of Cs-coated NiFe_2_O_4_ nanoparticles was performed, utilizing the obtained results for NiFe_2_O_4_ nanoparticles prepared by Bhosale et al.^55^. The previous study indicated that NiFe_2_O_4_ nanoparticles did not exhibit any antibacterial activity against *S. typhimurium*, *E. coli*, and *S. aureus* bacteria. Therefore, the coating of NiFe_2_O_4_ nanoparticles with Cs improved their antibacterial activity. Furthermore, the antibacterial activity of Cs-coated NiFe_2_O_4_ nanoparticles was compared with Cs-coated Fe_3_O_4_ nanoparticles prepared by El-Khawaga et al., and it was found that there was no significant difference in the obtained ZOI against *E. coli* bacteria^63^.

**Table 1:** MIC and MBC of Cs–NiFe_2_O_4_ NPs against *E. coli* and *S. aureus*.

Bacterial strain	MIC (mg/mL)	MBC (mg/mL)
*E. coli*	256	512
*S. aureus*	128	256

**Table 2:** Effect of Cs–NiFe_2_O_4_ NP loading dose on the antimicrobial activity against *E. coli* and *S. aureus*.

Bacterial strain/dose	Zone of inhibition (mm)						
	8 mg/mL	16 mg/mL	32 mg/mL	64 mg/mL	128 mg/mL	256 mg/mL	512 mg/mL
*E. coli*	–	–	–	–	–	18 ± 0.7	20 ± 1.2
*S. aureus*	–	–	–	–	18 ± 0.9	19 ± 0.1	23 ± 1.8

### Antioxidant activities

**Figure 7:**
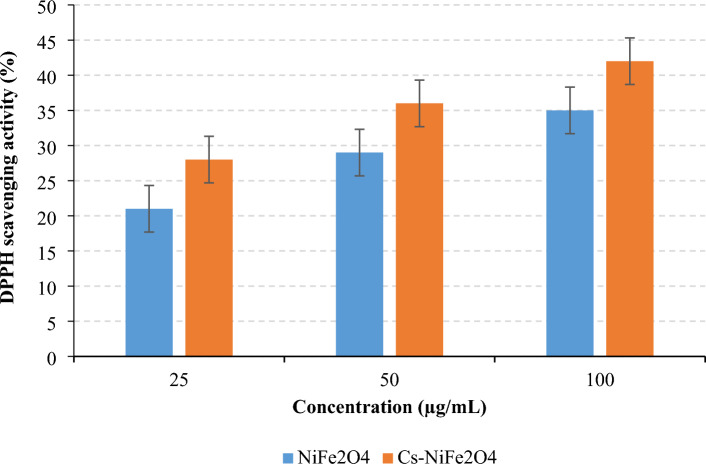
Antioxidant activity of NiFe_2_O_4_ NPs and Cs–NiFe_2_O_4_ NPs.

Lipid oxidation is one of the most critical factors in food spoilage and reduced shelf life due to decreased nutritional value and texture/flavor deterioration^64^. The antioxidant activity of synthesized NPs was investigated using the DPPH method (Fig. 7). The samples exerted considerable DPPH scavenging capacity. It can be attributed to the potential of nanoparticles to supply hydrogen for quenching DPPH radicals^65^. Another explanation is to transfer electrons from the nanoparticles’ oxygen atom to the DPPH nitrogen atom^66^. Several researches have also demonstrated the ability of antioxidant metal ions to neutralize free radicals^65,66^. The antioxidant potential was incremented after coating by chitosan; however, no significant differences were observed between the two nanoparticles at the same concentration (*P* ˃ 0.05). The antioxidant activity of uncoated and chitosan-coated NPs gradually enhanced from ~ 21% and ~ 28% to 35% and ~ 42%, respectively, by increasing NP concentration from 25 µg/mL to 100 µg/mL. A similar trend was reported by Kanagesan et al.^38^ for CuFe_2_O_4_ and ZnFe_2_O_4_ nanoparticles; however, the last nanoparticles conferred considerably inferior antioxidant ability compared to our developed nanoparticles at the same concentrations. The results of our research indicated that NiFe_2_O_4_ and Cs–NiFe_2_O_4_ NPs can be utilized as novel antioxidants in the food industry. Several mechanisms have been proposed for the antioxidant activity of Cs and its derivatives: (i) chitosan contains amino groups that can donate electrons, making it capable of scavenging free radicals, leading to protecting cells from oxidative stress^67^, (ii) Cs can bind to metal ions, such as copper and iron, inhibiting the production of harmful reactive oxygen species and reducing oxidative damage^68^, and (iii) it inhibits the activity of enzymes involved in the production of reactive oxygen species through blocking these enzymes^67,69^. Upon the formation of Cs–NiFe_2_O_4_ NPs, a reduction in the size of Cs coatings on the surface of NiFe_2_O_4_ NPs results in a notable increase in the exposed surface area of Cs. This phenomenon significantly enhances the affinity of Cs for interacting with free radicals, metal ions, and enzymes, consequently bolstering its inherent antioxidant activity. The comparison of antioxidant activity between Cs–NiFe_2_O_4_ nanoparticles and Ni-Zn ferrite nanoparticles prepared by Mondal et al. revealed an enhanced antioxidant activity in Cs–NiFe_2_O_4_ NPs when compared to Ni-Zn ferrite nanoparticles^70^. Furthermore, the Cs–NiFe_2_O_4_ composite demonstrated higher antioxidant activity than polyethylene glycol-capped nickel cobalt ferrite nanocomposites, as evaluated by Batool et al., suggesting that Cs significantly enhances the composite's antioxidant properties^71^.

## Conclusion

This study harnessed an efficient and cost-effective green synthesis approach, the co-precipitation technique, to fabricate chitosan-coated NiFe_2_O_4_ nanoparticles (Cs–NiFe_2_O_4_ NPs). A comprehensive characterization via XRD, FTIR, SEM, TEM, and VSM unequivocally demonstrated the successful chitosan coating of NiFe_2_O_4_ NPs. The compelling evidence from XRD patterns and FTIR spectra elucidated the intricate electrostatic and chemical interactions underpinning this coating process. Furthermore, the SEM analysis unveiled the remarkable transformation of surface morphology, rendering the coated nanoparticles remarkably smoother and more uniform than their uncoated counterparts. Notably, these synthesized nanoparticles displayed pronounced antioxidant properties, highlighting their potential to mitigate oxidative stress.

Their formidable antibacterial efficacy was equally impressive, as evidenced by their substantial inhibitory effect against *E. coli* and *S. aureus*. This pronounced antibacterial activity positions Cs–NiFe_2_O_4_ NPs as a compelling disinfection agent for addressing water contamination issues.

As a result, the multifaceted properties of Cs–NiFe_2_O_4_ NPs position them as promising materials with vast application potential. Their utility spans across various domains, including industrial, biological, packaging, and agricultural sectors. With their antibacterial and antioxidant attributes, these nanoparticles hold significant promise for enhancing a wide array of applications, such as water treatment and purification to eliminate harmful bacteria and contaminants from water sources, food packaging to help extend the shelf life of perishable food products by inhibiting bacterial growth and oxidative degradation, agriculture to protect crops from bacterial infections, and cosmetics and skincare to help in combating skin aging caused by oxidative stress and to provide antioxidant benefits and protection against skin-damaging free radicals.

## Data Availability

The datasets generated and analyzed during the current study were available from the corresponding author on reasonable request.
